# Notes on Use of Salol in the Treatment of Gout

**Published:** 1890-02

**Authors:** Wm. Perrin Nicolson

**Affiliations:** Atlanta, Ga.


### NOTES ON USE OF SALOL IN THE TREATMENT OF GOUT.

BY WM. PERRIN NICOLSON, M. D., ATLANTA, GA.

The use of the salicylic acid preparations for the relief of acute attacks of gout is well known, but my attention has not been called to the employment of its more agreeable compound, salol. Having no irritating effect on the stomach, and being free from the unpleasant nervous symptoms produced by the other compounds, it seems eminently applicable for the treatment of this very painful disease. The following notes of a few cases illustrate its action:

Case 1.—Mr. K., aged 36, a high liver. Father gouty, and personal history inducive of the disease. Attack in the left tarsus in June 1889, caused by sleeping with the window of car raised, and wind blowing on the foot. Local swelling with much tenderness and exquisite pain of characteristic nature.

Salol in doses of five grains every hour gave relief of the pain in a few hours, and on the second day the local trouble was sufficiently overcome for him to be at his office. He complained of slight deafness at one time during the treatment. There was no return of the disease.

Case II.—Mr. J. D. Y., aged 50, a high liver, and entitled by inheritance to gout. Has had two attacks before, each of about two weeks duration. After drinking some old Burgundy for dinner, in less than three hours felt a severe pain at a point just below the patella, where the preceding attacks had appeared. In a short time all of the usual symptoms of acute gout were manifest. Salol, five grains every hour gave entire relief of pain in a few hours, and the next morning he was able to leave his room. In two days he was well.

Case III.—Mr. J. C. R., aged 40. History of gout in the father. Two weeks before I saw him he had passed through an attack of nearly three weeks duration, which, after the trying of many remedies was finally relieved by the use of the salicylates. At the time of my visit he was feeling like a return of his former attack. I advised the use of salol, but he did not take it regularly, and the next morning I was called at an early hour, to find him in great agony, with the foot elevated at an angle of almost 90 deg., by the use of an inverted chair on which it rested. There was an angry swelling over the metatarso-phalangeal joint, with great tendernpss. There was so much suffering that it was necessary to administer a dose of morphia subcutaneously, and then the regular hourly dose of the salol was commenced. At my call in the evening all pain had disappeared, and the patient was perfectly comfortable except as to the tenderness left in the joint. On the second day he came to his office, wearing a slipper. As experience has proved best in all cases where the salicylates are used, a pill about three times daily was continued for a few days to prevent a relapse. This I recommend in all cases, no matter how thorough the relief.

---

Dr. W. F. Westmoreland, of this city, had a patient to die from the effects of ether, while operating for Fistula in Ano about ten days ago. He will give a detailed account of it in our next issue.

